# Knowledge and perceptions of blood donors of the Western Cape Blood Services, South Africa, toward vaginal sample donation for biobanking

**DOI:** 10.3389/frph.2024.1446809

**Published:** 2024-11-27

**Authors:** Shantelle Claassen-Weitz, Brian Kullin, Elloise du Toit, Sugnet Gardner-Lubbe, Jo-Ann S. Passmore, Heather Jaspan, Anna-Ursula Happel, Greg Bellairs, Caroline Hilton, Anika Chicken, Kirsten Welp, Hannah Livingstone, Adrian Brink

**Affiliations:** ^1^ Division of Medical Microbiology, Department of Pathology, Faculty of Health Sciences, University of Cape Town, Cape Town, South Africa; ^2^ Division of Medical Virology, Department of Pathology, Faculty of Health Sciences, University of Cape Town, Cape Town, South Africa; ^3^ Faculty of Health Sciences, Institute of Infectious Disease & Molecular Medicine (IDM), University of Cape Town, Cape Town, South Africa; ^4^ Centre for Multi-dimensional Data Visualisation (MuViSU), Department of Statistics and Actuarial Science, Faculty of Economic and Management Sciences, Stellenbosch University, Stellenbosch, South Africa; ^5^ National Health Laboratory Services, Groote Schuur Hospital, Cape Town, South Africa; ^6^ Division of Immunology, Department of Pathology, Faculty of Health Sciences, University of Cape Town, Cape Town, South Africa; ^7^ Western Cape Blood Service, Cape Town, South Africa

**Keywords:** blood donors, microbiome, vaginal, vaginal donors, vaginal microbiota transplant

## Abstract

**Introduction:**

Depletion of *Lactobacillus* species and an overgrowth of anaerobes in the vaginal tract bacterial vaginosis (BV)], is associated with non-optimal reproductive health outcomes, and increased susceptibility to sexually transmitted infections (STIs). BV is currently treated with antibiotics, although these provide suboptimal cure levels and high recurrence rates. Vaginal microbiota transplantation (VMT), the transfer of vaginal fluid from healthy donors with an optimal vaginal microbiota to a recipient with BV, has been proposed as an alternative treatment strategy.

**Methods:**

Here, we investigated knowledge and perceptions of blood donors to the concept of an optimal vaginal microbiome and VMT via the Western Cape Blood Service (WCBS) clinics in Cape Town, South Africa, by a self-administered questionnaire.

**Results & discussion:**

Analysis of responses from 106 eligible women showed that 86% (91/106) would consider donating samples. Responses significantly associated with willingness to donate vaginal samples included: (1) belief that helping others outweighs the inconvenience of donating vaginal sample (*p* = 1.093e^−05^) and (2) prior knowledge of the concept of a healthy vaginal microbiome (*p* = 0.001). Most potential donors (59/91; 65%) were willing to receive a VMT themselves if needed. Participants who were unwilling to donate vaginal samples (15/106; 14%) indicated that vaginal sample collection would be unpleasant and/or embarrassing. The benefits of a collaboration with WCBS for this project include the naturally altruistic nature of blood donors, the constant in-flow of donors to WCBS clinics, and the infrastructure and logistical aspects in place. Data from this observational study highlight factors affecting the willingness of blood donors to become vaginal sample donors.

## Introduction

The human microbiota and its role in health and disease have been extensively studied over the past few decades (1, 2). Disruption in microbial dynamics has been associated with a range of pathological disorders (2, 3) and hence current research is focused on therapeutic strategies to restore the microbiota to improve human health (4). One such strategy includes the transfer of microbiota from healthy donors to patients, referred to as a microbiota transplantation (5, 6), which is used in the treatment of *Clostridioides difficile* infections with faecal microbiota transplantation (FMT) (7–10). Microbiome transplantation has also been explored for treatment of vaginal dysbioses (11).

Bacterial vaginosis (BV) has been linked to a range of adverse health outcomes, including pelvic inflammatory disease (PID), increased susceptibility to sexually transmitted infections (STIs), risk of complications during pregnancy and poor birth outcomes (12, 13). The current standard of care for treatment of BV is antibiotics, although these are often associated with high rates of recurrence within 6 months of treatment (14). “Resetting” the vaginal microbiome to a beneficial *Lactobacillus*-dominated bacterial community via vaginal microbiota transplantation (VMT) from donors who persistently maintain optimal *Lactobacillus*-dominant vaginal communities may improve effectiveness of treatment for clinically severe and recurrent BV (11, 15).

Despite the high prevalence of BV in sub-Saharan Africa, with rates as high as 60% in some populations (16), a national vaginal microbiome biobanks have not been established. The advantage of a regional biobank is the well-curated, extensively screened samples for fundamental proof-of-concept studies investigating the efficacy of VMT in the local setting. The consecutive collection of vaginal samples from individual women allows for the identification of microbial and host factors associated with longitudinally stable, optimal vaginal microbiotas (17), providing an invaluable resource of potential *Lactobacillus* strains to develop multi-strain live biotherapeutic products to stabilise optimal vaginal communities (18).

Blood donors have been identified as ideal donors for microbiome collections (19, 20). Collaborations with blood donor services in Europe and the United Kingdom have resulted in the establishment of biobanks for use in FMT. To establish a vaginal microbiome biobank in Africa, we investigated the interest and willingness amongst female blood donors in the Western Cape, South Africa, to donate vaginal samples. The primary aim of our study was to provide strategic data for key stakeholders towards actualisation of a biobank of vaginal microbiomes for the purpose of VMT. A secondary aim was to concurrently ascertain the social context, knowledge, and attitude of potential participation in establishing a vaginal microbiome biobank within South Africa.

## Methods

### Study design and data collection

Willingness of WCBS blood donors to donate vaginal samples for microbiome biobanking was investigated via a cross-sectional, questionnaire-based survey. The observational pilot study was conducted between 1 June 2022 and 1 July 2022 at three WCBS donation centres in Cape Town, South Africa spanning a total area of 106 km^2^. Inclusion criteria to be interviewed included being female, age (18–50 years) and willingness to provide informed consent. Anonymous blood donors who met the inclusion criteria were provided with infographics on vaginal sample donation, which included (i) reasons for donating vaginal samples for microbiome biobanking, (ii) donor eligibility, and (iii) the self-collected vaginal sample collection method. A Softdisc® vaginal disc (https://softdisc.com/), which can be used to collect vaginal secretions was demonstrated to female blood donors. After obtaining informed consent, respondents’ demographics, history of blood and organ donations, VMT-related knowledge and perceptions, modifiable aspects of vaginal sample donations, and primary reasons for becoming or not becoming a vaginal sample donor were collected via pre-populated paper-based questionnaires. We approached eligible female blood donors after blood donation. Compensation was not offered to participants in the survey and ethical approval was obtained from the University of Cape Town Human Research Ethics Committee before study commencement (HREC REF 122/2022).

### Data analysis

Anonymised demographic and interview data were aggregated for descriptive purposes and statistical analysis. Data from each questionnaire were captured in Microsoft Excel and checked by two other co-investigators for accuracy and completeness.

The variables considered in this investigation were divided into three categories. First, the variable “willing donor” was modelled as a function of the possible covariates (participant characteristics) listed in Supplementary Table S1. Next, variables pertaining specifically to willing donors were used to characterise the willing donors (Supplementary Table S2). Finally, reasons for being unwilling to donate vaginal sample were investigated (Supplementary Table S3). Participants were excluded if participants were <18 and >50 years of age, or if participants submitted incomplete questionnaires. In addition, records where the “willing donor” field was missing were excluded.

The random Forest package (21) in R software was used to identify variables which contribute to being a “willing donor”. A total of 500 classifications trees were built using random subsets of the covariates. Based on all 500 trees, a variable importance plot (VIP) was produced, indicating the importance of each covariate in classifying potential donors as willing or not. A logistic regression model was fitted for variables showing the largest mean decrease in accuracy. Variables were selected in a stepwise manner: (1) remove the variable with largest mean decrease in accuracy in the VIP and add it to a logistic regression model; (2) fit another random forest to produce a VIP; (3) repeat until variables added to the logistic regression are not significant for classification.

Variables used to characterise willing vaginal sample donors were investigated via multivariate analysis. The latter allowed to determine which response to a particular question more often corresponded to responses to other questions. A joint correspondence analysis was performed using the ca package (22) in R software. Subset correspondence analysis was selected to suppress the use of the “missing” categories in determining the plot while keeping the row totals constant. Proportions (indicated as pie slices in the plot) were computed based on the frequency of responses for each category in a specific variable.

## Results

A total of 115 female blood donors visiting one of three blood donation clinics in Cape Town, South Africa consented and completed the questionnaire. Of these, 6 participants were excluded <18 and >50 years of age (*n* = 3), incomplete questionnaires (*n* = 3)]. Of the 109 participants who completed the questionnaire, three records were excluded because the “willing donor” field was missing from the completed questionnaire. Overall, responses from 106 participants were included in the analysis.

### Participant characteristics

Most female blood donors who were included in the study were willing to donate vaginal samples (91/106, 86%) and indicated that helping others would outweigh any inconvenience vaginal sample donation may impose (89/106, 84%). Most women who provided responses regarding economic compensation for their vaginal sample donation indicated that compensation between 0 and 250 ZAR would be considered adequate (61%; 65/106), although 27% (29/106) did not provide a response for the section (Table 1). Participants who were willing to donate samples were primarily between the ages of 21 and 40 (62/106, 58%) and employed (69/106, 65%) (Table 1). Most participants did not have prior knowledge of the concept of a healthy vaginal microbiome (72/106, 68%), what VMT is (88/106, 83%), how VMTs could help other women (85/106, 80%), and how a vaginal microbiome can be sampled (86/106, 81%) (Table 1).

**Table 1:** Participant characteristics of blood donors surveyed as potential vaginal sample donors, *N* = 106.

Participant characteristics	*n* (%)
Site at which questionnaire was collected	
Blue Route Mall	23 (22)
Long Street	49 (46)
N1 City Mall	34 (32)
Participant age at visit	
18–21	19 (18)
21–30	34 (32)
31–40	28 (26)
41–50	25 (24)
Categorical classification of participant occupation	
Learner/student	25 (24)
Employed	69 (65)
Unemployed	8 (8)
Missing	4 (3)
Do you regularly donate blood?	
Yes	74 (70)
No	32 (30)
Are you an organ donor/would you consider becoming an organ donor?	
Yes	63 (59)
No	25 (24)
Unsure	18 (17)
Do you have prior knowledge of the concept of a healthy vaginal microbiome?	
Yes	28 (26)
No	72 (68)
Unsure	6 (6)
Do you have prior knowledge of what a VMT is?	
Yes	12 (11)
No	88 (83)
Unsure	6 (6)
Do you have prior knowledge of how VMTs could help patients?	
Yes	13 (12)
No	85 (80)
Unsure	8 (8)
Do you have prior knowledge of how a vaginal biome is sampled?	
Yes	20 (19)
No	86 (81)
Would you be more likely to become a vaginal sample donor if economic compensation is offered?	
Yes	58 (55)
No	47 (44)
Missing	1 (1)
Would you consider being a vaginal sample donor if receiving the following compensation per donation:	
None	5 (5)
≤150 ZAR (≤8 USD)	23 (22)
>150 to ≤250 ZAR (>8 to ≤13 USD)	42 (39)
>250 ZAR (>13 USD)	4 (4)
Any amount/travel costs	3 (3)
Missing	29 (27)
Do you believe helping other is more important than any inconvenience being a vaginal donor may impose?	
Yes	89 (84)
No	13 (12)
Missing	4 (4)

VMT, vaginal microbiota transplant.

*Willingness to become a vaginal sample donor by participant characteristics: Identifying variables which contribute to being a* “*willing donor*”.

**Figure 1:**
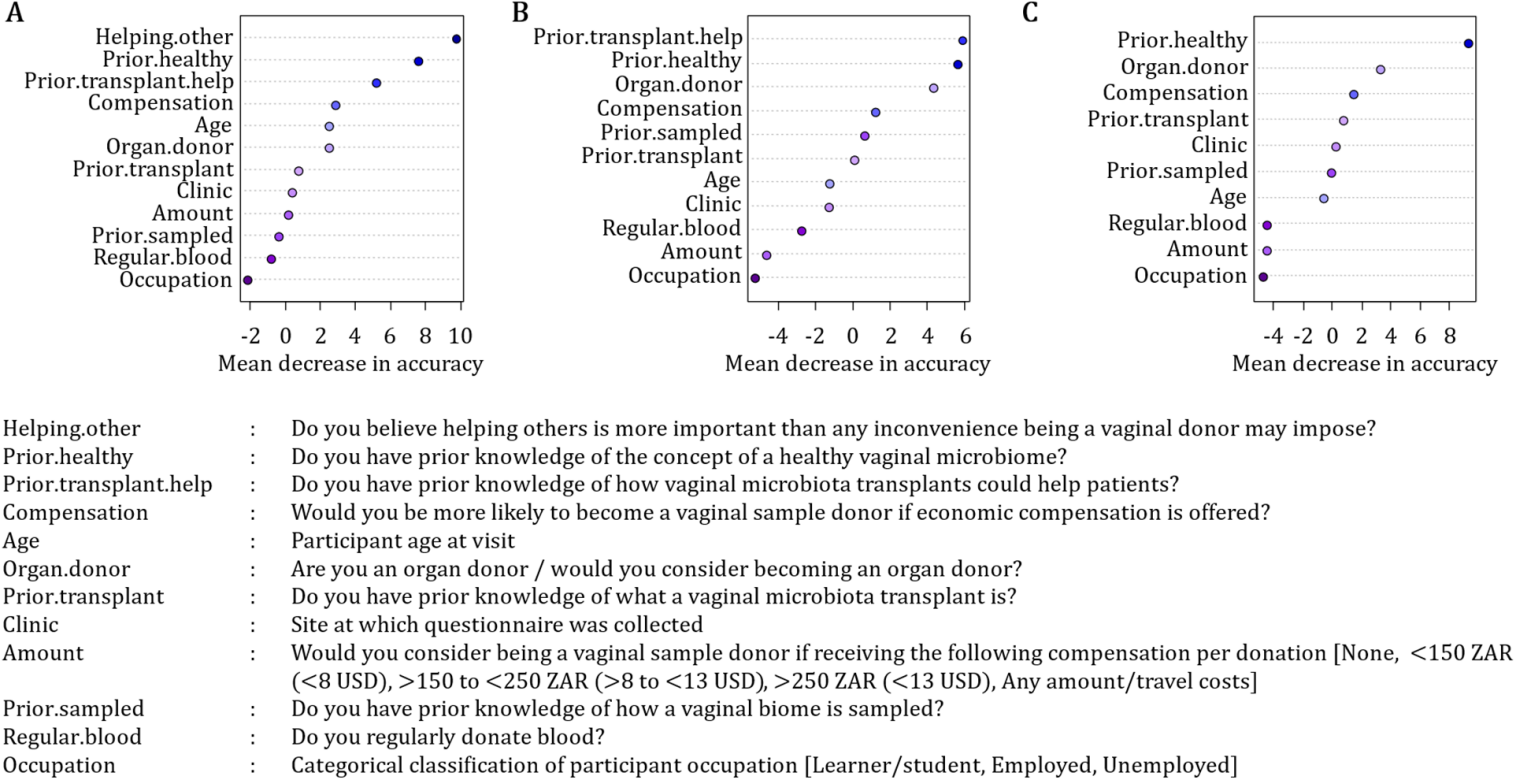
Variance importance plots showing important covariates in accurately classifying potential donors as willing donors. **(A)** The largest mean decrease in accuracy was found with the removal of “opinions as to whether helping other outweighs the inconvenience of donation”, showing that it plays the most important role in accurately classifying potential donors as willing or not. Removing “opinions as to whether helping other outweighs the inconvenience of donation” **(B)**, or both “opinions as to whether helping other outweighs the inconvenience of donation” and “prior knowledge of the concept of a healthy vaginal microbiome” **(C)** from the data lead to different results when applying the random forest method, confirming that the results are obtained by chance. Some of the covariates that appeared as important are compensation, “being/considering becoming an organ donor” and “prior knowledge of what a VMT is”.

Variable importance plots identified benefit outweighs inconvenience beliefs, followed by prior knowledge of the concept of a healthy vaginal microbiome, and prior knowledge of how VMTs could help patients, as the variables with the most important role in accurately classifying potential donors as willing or not (Figure 1A). Other important covariates were compensation, being/considering becoming an organ donor and prior knowledge of what a VMT is (Figures 1A–C).

A logistic regression model with benefit outweighs inconvenience beliefs, prior knowledge of the concept of a healthy vaginal microbiome, and prior knowledge of how VMTs could help patients showed that benefit outweighs inconvenience beliefs (*p* = 1.093e^−05^) and prior knowledge of the concept of a healthy vaginal microbiome (*p* = 0.001) were covariates significantly associated with willingness to donate vaginal samples.

The non-significant *p*-value for the variable prior knowledge of how VMTs could help patients is the result of collinearity in the data (i.e., high correlation between prior knowledge of the concept of a healthy vaginal microbiome and prior knowledge of how VMTs could help patients). Overall, a higher proportion of women (26%) had prior knowledge of a heathy vaginal microbiome than prior knowledge of how VMTs could help women with BV (12%), although most women (63%) did not have prior knowledge of either. As these variables are both categorical, a Pearson's chi-square test of association was performed. A stronger relationship was observed for diagonal combinations (no-no, yes-yes and unsure-unsure) compared to the off-diagonal combinations (Pearson chi-square test *p*-value: < 0.0001). These data suggest that either of these variables (prior knowledge of the concept of a healthy vaginal microbiome and prior knowledge of how VMTs could help patients) could be used to predict VMT donor willingness.

Fitting a logistic regression model with benefit outweighs inconvenience beliefs and prior knowledge of the concept of a healthy vaginal microbiome showed women who believed that benefit outweighs inconvenience are 50% more likely to be willing vaginal sample donors than those who do not believe this (odds ratio OR]: 1.507, 95% confidence interval CI]: 1.263–1.797, *p* < 0.001). Participants with prior knowledge of the concept of a healthy vaginal microbiome are only 9% more likely to be willing vaginal sample donors compared to participants without prior knowledge (OR: 1.094, 95% CI: 0.957–1.249, *p* < 0.001). Fitting a logistic regression model with benefit outweighs inconvenience beliefs and prior knowledge of how VMTs could help patients showed that women who believed that benefit outweighs inconvenience are 60% more likely to be willing vaginal sample donors compared to participants who do not (OR: 1.594, 95% CI: 1.327–1.917, *p* < 0.001). Women with prior knowledge of how VMTs could help patients were only 7% more likely to be willing vaginal sample donors compared to participants without prior knowledge (OR: 1.070, 95% CI: 0.890–1.286, *p* = 0.019). The logistic regression model including the variables benefit outweighs inconvenience beliefs and prior knowledge of the concept of a healthy vaginal microbiome provides a slightly more accurate prediction of willing donors (87.7%) compared to the model including variables benefit outweighs inconvenience beliefs and prior knowledge of how a VMT can help patients (86.8%). More women had prior knowledge of what a healthy vaginal microbiome was than those who have prior knowledge of how a VMT can help patients.

### Characteristics of willing vaginal sample donors

**Figure 2:**
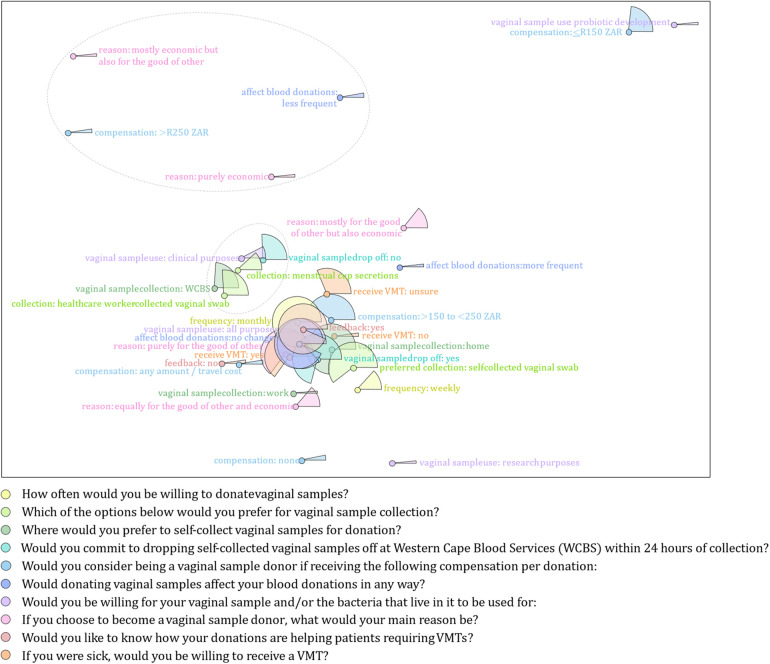
Multivariate analysis of variables used to characterize willing vaginal sample donors. Each variable (and its categories) used to characterize willing vaginal sample donors is represented using a unique colour. The frequency of responses for each category in a specific variable are shown as proportions (represented as pie slices in the plot). Category levels that appear close by, tend to appear together in responses while category levels that appear far apart, typically belong to different participants.

Of the 106 women included in this study, 91/106 (86%) of women indicated that they would donate vaginal samples or that their vaginal sample and/or bacteria may be used for any purpose. Most potential vaginal sample donors would be willing to donate monthly (75/91, 83%) as opposed to weekly (13/91, 14%); would prefer to self-collect vaginal samples (55/91, 89%) at home (66/91, 73%), rather than at the WCBS clinics (22/91, 24%); and would commit to dropping self-collected vaginal samples off at the WCBS HQ within 24 h of collection (65/91, 71%) (Table 2). Most willing vaginal sample donors (58/91, 64%) would consider vaginal sample donations if up to 250 ZAR was offered as compensation, whilst 26% omitted this information. Most willing vaginal sample donors indicated that donating vaginal samples would not affect blood donation frequencies (85/91, 94%) and that their vaginal samples and/or the bacteria could be used for both clinical and research purposes (80/91, 88%). Most potential donors indicated that they would donate purely for the good of others (61/91, 67%), and more than 90% reported that they would appreciate feedback on how their donations helped patients requiring VMTs. Fifty-nine (65%) potential donors expressed a willingness to receive a VMT themselves if needed, although 32% were unsure (Table 2; Figure 2).

**Table 2:** Participant characteristics of willing vaginal sample donors, *N* = 91.

Participant characteristics	*n* (%)
How often would you be willing to donate stool?	
Weekly	13 (14)
Monthly	75 (83)
Missing	3 (3)
Which of the options below would you prefer for vaginal sample collection?	
Self-collected vaginal swab	55 (89)
Healthcare worker-collected vaginal swab	24 (8)
Menstrual cup secretions (e.g., Softcup)	11 (3)
Missing	1 (1)
Where would you prefer to self-collect vaginal samples for donation?	
Home	66 (73)
WCBS	22 (24)
Work	2 (2)
Missing	1 (1)
Would you be able to commit to dropping self-collected vaginal sample off at the WCBS HQ, Pinelands, Cape Town within 24 h of collection?	
Yes	65 (71)
No	24 (27)
Missing	2 (2)
Would you consider being a vaginal sample donor if receiving the following compensation per donation	
None	4 (4)
≤150 ZAR (≤8 USD)	21 (23)
>150 to ≤250 ZAR (>8 to ≤13 USD)	37 (41)
>250 ZAR (>18 USD)	3 (3)
Any amount/travel costs	3 (3)
Missing	23 (26)
Would donating vaginal samples affect your blood donations in any way?	
No changes to blood donations	85 (94)
More frequent blood donations	2 (2)
Less frequent blood donations	3 (3)
Missing	1 (1)
Would you be willing for your vaginal sample and/or the bacteria that live in it to be used for	
Clinical purposes	8 (9)
Research purposes	2 (2)
Development of probiotic products	1 (1)
All the above	80 (88)
If you choose to become a vaginal sample donor, what would your main reason be?	
Purely for the good of others	61 (67)
Mostly for the good of others but also economic	14 (16)
Equally for the good of others and economic	13 (14)
Mostly economic but also for the good of others	1 (1)
Purely economic	1 (1)
Missing	1 (1)
Would you like to know how your donations are helping patients requiring vaginal microbiota transplants?	
Yes	85 (94)
No	3 (3)
Missing	3 (3)
If you were sick, would you be willing to receive a VMT?	
Yes	59 (65)
Unsure	29 (32)
No	3 (3)

WCBS, Western Cape Blood Service; VMT, vaginal microbiota transplant.

The joint correspondence analysis showed which response to a particular question more often corresponded to responses to other questions: Most willing vaginal sample donors agree on self-collection using a vaginal swab at home at monthly intervals, would commit to dropping off vaginal samples within 24 h of collection and would donate purely for the good of others without blood donation frequencies being compromised. Most willing donors would also like feedback on how their donations are helping patients, are open to their vaginal samples to be used for all purposes and would receive a VMT if needed (Figure 2). Interestingly, participants who would not want to receive a VMT responded similarly with participants who would want to receive a VMT (Figure 2). Since willing vaginal sample donors made up approximately 86% of participants overall (91/106) and as a group provided 75% or more of participant responses to most questions (Figure 2), we can estimate that approximately 65% of all participants shared these views.

Willing vaginal sample donors who would consider being a donor if they received larger amounts of compensation (>R250 ZAR) would donate vaginal samples primarily for economic reasons purely economic; mostly economic but also for the good of others]. Interestingly, for this group of participants, vaginal sample donations would result in less frequent blood donations (Figure 2).

Participants who would not commit to dropping off vaginal samples within 24 h of collection would prefer collection to take place at WCBS clinics via menstrual cup collection or healthcare worker-collected vaginal swabs as opposed to self-collected at home or work using vaginal swabs (Figure 2). The latter group of participants are also more willing for their vaginal sample and/or the bacteria that live in it to be used for clinical purposes as opposed to research purposes, probiotic development, or all purposes.

### Reasons for being unwilling to donate vaginal sample

Each of the unwilling vaginal sample donors (*N* = 15) provided one or more reasons for their response. The primary reason was that collection would be unpleasant (36%) or embarrassing (14%). Other reasons for being unwilling to donate were that it would be too much of a commitment to donate weekly or monthly (14%), that medical examinations at the WCBS clinic during donations would be too time consuming or exhaustive (14%), that collection procedure seems too complicated (9%), collection would not align with cultural beliefs (9%), and logistics (5%). None of the unwilling vaginal donors disagreed with the concept of VMT procedures. Most unwilling vaginal sample donors indicated that they were unsure (60%) or not be willing (27%) to receive a VMT if needed.

## Discussion

Globally, the burden of BV and associated sequelae including increased risk of acquisition of HIV and other STIs, PID and adverse pregnancy outcomes is high (23). Notably, women in resource-poor settings, particularly in sub-Saharan Africa, are most affected by BV (16).

Despite the high prevalence of BV (24), and lack of long-term therapeutic strategies, there has not been a fundamentally new therapy for BV in decades. VMT has shown promise in small scale pilot studies, suggesting the need for larger studies in more diverse populations (15, 25). However, an ongoing challenge is the identification of suitable donors since the extremely stringent screening procedures associated with the therapeutic use of VMTs result in significant screen failure (26). Furthermore, a better understanding of the different types of vaginal microbiomes and their association with BV, as well as factors influencing community stability, are important in managing the condition. The latter requires access to longitudinally collected stored samples. Successful collection, screening, and storage of vaginal fluid from healthy donors in a vaginal microbiome biobank is the first step toward enabling both goals. This observational study highlights the potential of recruiting blood donors as vaginal sample donors, previously reported for stool sample donations (19, 20).

Most participants interviewed at the WCBS clinics were willing to become vaginal sample donors. This was surprising as most participants displayed limited prior knowledge of the concept of a healthy vaginal microbiome, VMTs, the potential benefits of VMTs, and processes involved to sample the vaginal biome. When investigating variables which contribute to being a “willing donor”, we found that the major driver for future donations would be to help others. This could be ascribed to the general altruistic nature of blood donors, but also may represent a slightly biased sample as they were also women who were willing to participate in this research. In addition, we found that prior knowledge of the concept of a healthy vaginal microbiome further contributed to being a “willing donor”. Hence, awareness-raising campaigns and promoting the benefits of vaginal sample donations for recipients would provide strong motivation for vaginal sample donations among an already altruistic group of blood donors.

Given the altruistic nature of the participants, it is not surprising that most willing vaginal sample donors expressed that they would appreciate feedback on how their donations are helping women with BV. These women also agreed that their vaginal samples could be used for both clinical and research purposes. Most willing vaginal sample donors also indicated that they would want to receive a VMT if needed. The latter suggests that these donors are of the opinion that VMTs could truly benefit the recipients, which again emphasises their altruistic nature.

Interestingly, we found that participants who agreed to donate vaginal samples primarily for economic reasons, also indicated that these would result in them donating blood less frequently. The latter disagrees with previous reports of potential stool donors indicating that they would primarily donate for economic reasons and that stool donation would not affect their blood donation frequency (27, 28). The latter suggest that motivations for donating these two distinct sample types likely differ.

Most participants reported that they would prefer to donate monthly, rather than weekly. Donors in previous VMT trials typically provided samples at a much higher frequency (usually 14–20 samples usually over a period of ∼40 days) (17, 25). The reason for the high frequency of collection in therapeutic trial settings is likely to maximise sample collection during the relatively short period that donors could be expected to adhere to the lifestyle restrictions involved in the donation of samples for therapeutic use (complete sexual abstinence, no blood donations, etc.). Participants who would only be willing to donate less frequently could, nevertheless, still be a valuable source of samples for longer term longitudinal studies.

Primary reasons for being unwilling to donate vaginal samples included the unpleasant and/or embarrassing experience these donations would accompany. This observation was also made when interviewing blood donors for stool sample donations (27, 29). Awareness-raising campaigns around vaginal sample donation may be necessary to encourage VMT donations. Building confidence in the concept of VMT in female donors will have to involve critical and clear communication, education campaigns and strategic advertising, detailing the process of vaginal sample collection from start to finish and sharing previous donors’ testimonies.

This observational study has been important in highlighting the possible ambivalence and reasons therefore, in women donors to participate in a vaginal sample biobank. This information will allow us to carefully design strategies to address and resolve these concerns so that women donors feel more confident to become vaginal sample donors. It is important that a database of continuous donors with sustained participation is established for two reasons: (1) the costs involved in screening potential vaginal sample donors are significant, and (2) to build a feasible process to start a vaginal sample biobank for VMT in South Africa.

One important limitation of the current study is that it did not investigate whether potential donors would be willing to adhere to the stringent lifestyle restrictions involved in becoming donors for VMT purposes. Follow up discussions, including full descriptions of inclusion criteria and structured interviews with a smaller focus group recruited at one of the blood donation clinics would provide insights into what proportion of participants would be suitable. Another limitation of this study is that the questionnaire did not include information or questions regarding the changes in vaginal microbiome around and during the menstrual cycle. In follow up discussions, it would be made clear that VMT donations would not be requested during the menstrual phase.

To conclude, our data has indicated that the City of Cape Town in the Western Cape, South Africa, is a feasible option to start a vaginal microbiome biobank, with most female blood donors being open to the concept. This investigation should be extended into other areas of South Africa to confirm that willingness to participate in a vaginal microbiome biobank is the same in the rest of the country. In addition, doing this in collaboration with the WCBS provides an accessible and sustainable source of potential donors to meet the continuous needs of a future vaginal microbiome biobank.

## Data Availability

The datasets presented in this study can be found in online repositories. The names of the repository/repositories and accession number(s) can be found in the article/Supplementary Material.

## References

[1] Berg G Rybakova D Fischer D Cernava T Vergès M-CC Charles T Viruses in the built environment (VIBE) meeting report. Microbiome. (2020) 8(1):1–22. 10.1186/s40168-019-0777-4 31901242 PMC6942371

[2] Manos JIM. The human microbiome in disease and pathology. APMI. (2022) 130(12):690–705. 10.1111/apm.13225 PMC9790345 35393656

[3] Vijay A Valdes AM. Role of the gut microbiome in chronic diseases: a narrative review. Eur J Clin Nutr. (2022) 76:489–501. 10.1038/s41430-021-00991-6 34584224 PMC8477631

[4] Young VB. The role of the microbiome in human health and disease: an introduction for clinicians. Br Med J. (2017) 356:j831. 10.1136/bmj.j831 28298355

[5] Junca H Pieper DH Medina E. The emerging potential of microbiome transplantation on human health interventions. Comput Struct Biotechnol J. (2022) 20:615–27. 10.1016/j.csbj.2022.01.009 35140882 PMC8801967

[6] Zhang F Cui B He X Nie Y Wu K Fan D Microbiota transplantation: concept, methodology and strategy for its modernization. Protein Cell. (2018) 9(5):462–73. 10.1007/s13238-018-0541-8 29691757 PMC5960466

[7] Debast SB Bauer MP Kuijper EJ. European Society of Clinical Microbiology and Infectious Diseases: update of the treatment guidance document for clostridium difficile infection. Clin Microbiol Infect. (2014) 20(Suppl. 2):1–26. 10.1111/1469-0691.12418 24118601

[8] National Institute for Health and Care Excellence (NICE). Faecal Microbiota Transplant for Recurrent Clostridium difficile Infection. United Kingdom: Interventional Procedures Guidance. (2014). Available online at: https://www.nice.org.uk/guidance/ipg485

[9] Sokol H Galperine T Kapel N Bourlioux P Seksik P Barbut F Faecal microbiota transplantation in recurrent Clostridium difficile infection: Recommendations from the French Group of Faecal microbiota Transplantation. Dig Liver Dis. (2016) 48:242–7. 10.1016/j.dld.2015.08.017 26433619

[10] Trubiano JA Cheng AC Korman TM Roder C Campbell A May MLA Australasian Society of Infectious Diseases updated guidelines for the management of clostridium difficile infection in adults and children in Australia and New Zealand. Intern Med J. (2016) 46(4):479–93. 10.1111/imj.13027 27062204

[11] Delong K Zulfiqar F Hoffmann DE Tarzian AJ Ensign LM. Vaginal microbiota transplantation: the next frontier. J Law Med Ethics. (2019) 47(4):555–67. 10.1177/1073110519897731 31957577

[12] Arnold KB Burgener A Birse K Romas L Dunphy LJ Shahabi K Increased levels of inflammatory cytokines in the female reproductive tract are associated with altered expression of proteases, mucosal barrier proteins, and an influx of HIV-susceptible target cells. Mucosal Immunol. (2016) 9(1):194–205. 10.1038/mi.2015.51 26104913

[13] Ness RB Kip KE Hillier SL Soper DE Stamm CA Sweet RL A cluster analysis of bacterial vaginosis-associated microflora and pelvic inflammatory disease. Am J Epidemiol. (2005) 162(6):585–90. 10.1093/aje/kwi243 16093289

[14] Mtshali A San JE Osman F Garrett N Balle C Giandhari J Temporal changes in vaginal microbiota and genital tract cytokines among South African women treated for bacterial vaginosis. Front Immunol. (2021) 12:1–13. 10.3389/fimmu.2021.730986 PMC8477043 34594336

[15] Lev-Sagie A Goldman-Wohl D Cohen Y Dori-Bachash M Leshem A Mor U Vaginal microbiome transplantation in women with intractable bacterial vaginosis. Nat Med. (2019) 25(10):1500–4. 10.1038/s41591-019-0600-6 31591599

[16] Jespers V Crucitti T Menten J Verhelst R Mwaura M Mandaliya K. Prevalence and correlates of bacterial vaginosis in different sub-populations of women in sub-Saharan Africa: a cross-sectional study. PLoS Negl Trop Dis. (2014) 9(10):e109670. 10.1371/journal.pone.0109670 PMC4188821 25289640

[17] Yockey LJ Hussain FA Bergerat A Reissis A Worrall D Xu J Screening and characterization of vaginal fluid donations for vaginal microbiota transplantation. Sci Rep. (2022) 12(1):1–9. 10.1038/s41598-022-22873-y 36289360 PMC9606370

[18] Ma B France MT Crabtree J Holm JB Humphrys MS Brotman RM A comprehensive non-redundant gene catalog reveals extensive within-community intraspecies diversity in the human vagina. Nat Commun. (2020) 11:940. 10.1038/s41467-020-14677-3 32103005 PMC7044274

[19] Gayet-Ageron A Rudaz S Perneger T. Biobank attributes associated with higher patient participation: a randomized study. Eur J Hum Genet. (2017) 25(1):31–6. 10.1038/ejhg.2016.132 PMC5159767 27703145

[20] Raivola V Snell K Helén I Partanen J. Attitudes of blood donors to their sample and data donation for biobanking. Eur J Hum Genet. (2019) 27(11):1659–67. 10.1038/s41431-019-0434-1 31147625 PMC6871534

[21] Liaw A Wiener M. Classification and regression by Random forest. R News. (2002) 2(3):18–22.

[22] Nenadic O Greenacre M. Correspondence analysis in R, with two- and three-dimensional graphics: the ca package. J Stat Softw. (2007) 20(3):1–13.

[23] van de Wijgert JHHM Jespers V. The global health impact of vaginal dysbiosis. Res Microbiol. (2017) 168(9–10):859–64. 10.1016/j.resmic.2017.02.003 28257809

[24] Kenyon C Colebunders R Crucitti T. The global epidemiology of bacterial vaginosis: a systematic review. Am J Obstet Gynecol. (2013) 209(6):505–23. 10.1016/j.ajog.2013.05.006 23659989

[25] Wrønding T Vomstein K Bosma EF Mortensen B Westh H Heintz JE Antibiotic-free vaginal microbiota transplant with donor engraftment, dysbiosis resolution and live birth after recurrent pregnancy loss: a proof of concept case study. EClinicalMedicine. (2023) 61:102070. 10.1016/j.eclinm.2023.102070 37528843 PMC10388571

[26] Delong K Bensouda S Zulfiqar F Zierden HC Hoang TM Abraham AG Conceptual design of a universal donor screening approach for vaginal microbiota transplant. Front Cell Infect Microbiol. (2019) 9:1–16. 10.3389/fcimb.2019.00306 31555606 PMC6722226

[27] Claassen-Weitz S Du Toit E Gardner-Lubbe S Kullin B Bellairs G Hilton C Knowledge and perceptions of South African blood donors towards biobanking and stool donation. S Afr J Infect Dis. (2024) 39(1):645. 10.4102/sajid.v39i1.645 39507519 PMC11538471

[28] Mcsweeney B Allegretti JR Fischer M Xu H Goodman J Monaghan T In search of stool donors: a multicenter study of prior knowledge, perceptions, motivators, and deterrents among potential donors for fecal microbiota transplantation. Gut Microbes. (2020) 11(1):51–62. 10.1080/19490976.2019.1611153 31122134 PMC6973337

[29] Jørgensen SMD Erikstrup C Dinh KM Lemming LE Dahlerup JF Hvas CL. Recruitment of feces donors among blood donors: results from an observational cohort study. Gut Microbes. (2018) 9(6):540–50. 10.1080/19490976.2018.1458179 29617178 PMC6287698

