# Supplementary material for: Knowledge and perceptions of blood donors of the Western Cape Blood Services, South Africa, toward vaginal sample donation for biobanking
Source: Front Reprod Health. 2024 Nov 27;6:1446809. doi: 10.3389/frph.2024.1446809 (PMC11631888; doi:10.3389/frph.2024.1446809)
Supplement: Supplementary file 1 [file Datasheet1.pdf]

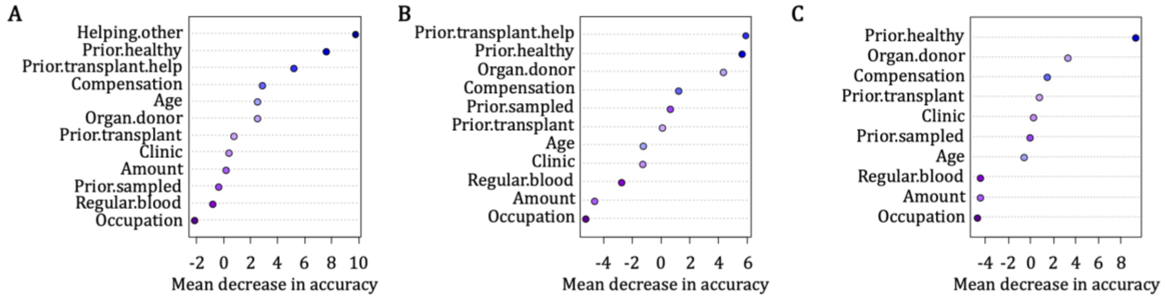

Helping.other	:	Do you believe helping others is more important than any inconvenience being a vaginal donor may impose?
Prior.healthy	:	Do you have prior knowledge of the concept of a healthy vaginal microbiome?
Prior.transplant.help	:	Do you have prior knowledge of how vaginal microbiota transplants could help patients?
Compensation	:	Would you be more likely to become a vaginal sample donor if economic compensation is offered?
Age	:	Participant age at visit
Organ.donor	:	Are you an organ donor / would you consider becoming an organ donor?
Prior.transplant	:	Do you have prior knowledge of what a vaginal microbiota transplant is?
Clinic	:	Site at which questionnaire was collected
Amount	:	Would you consider being a vaginal sample donor if receiving the following compensation per donation [None, <150 ZAR (<8 USD), >150 to <250 ZAR (>8 to <13 USD), >250 ZAR (<13 USD), Any amount/travel costs]
Prior.sampled	:	Do you have prior knowledge of how a vaginal biome is sampled?
Regular.blood	:	Do you regularly donate blood?
Occupation	:	Categorical classification of participant occupation [Learner/student, Employed, Unemployed]

Figure 1. Variance importance plots showing important covariates in accurately classifying potential donors as willing donors.

- A) The largest mean decrease in accuracy was found with the removal of 'opinions as to whether helping other outweighs the inconvenience of donation', showing that it plays the most important role in accurately classifying potential donors as willing or not. Removing 'opinions as to whether helping other outweighs the inconvenience of donation' (B), or both 'opinions as to whether helping other outweighs the inconvenience of donation' and 'prior knowledge of the concept of a healthy vaginal microbiome' (C) from the data lead to different results when applying the random forest method, confirming that the results are obtained by chance. Some of the covariates that appeared as important are compensation, 'being / considering becoming an organ donor' and 'prior knowledge of what a VMT is'.

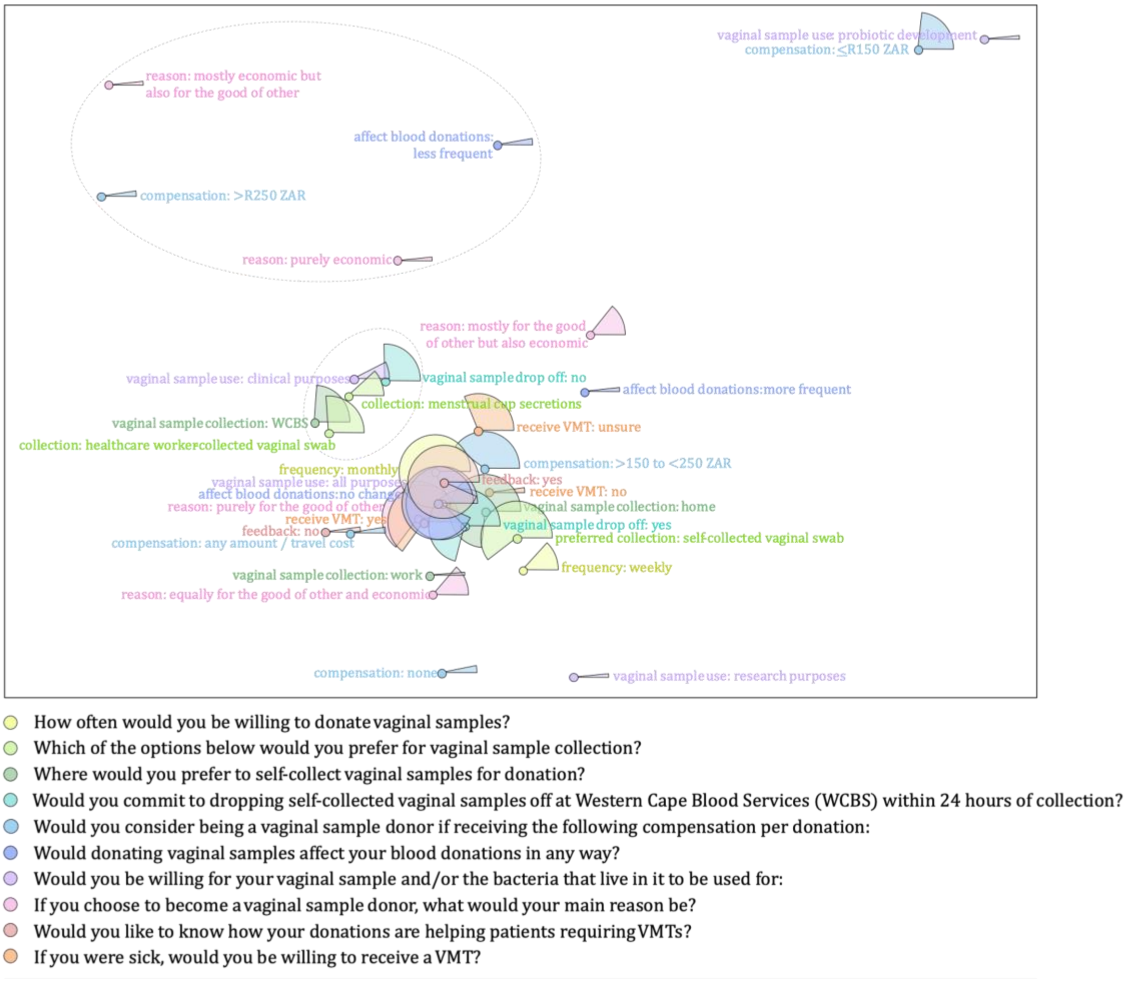

Figure 2. Multivariate analysis of variables used to characterize willing vaginal sample donors.

Each variable (and its categories) used to characterize willing vaginal sample donors is represented using a unique colour. The frequency of responses for each category in a specific variable are shown as proportions (represented as pie slices in the plot). Category levels that appear close by, tend to appear together in responses while category levels that appear far apart, typically belong to different participants.
