# Supplementary material for: Knowledge and perceptions of blood donors of the Western Cape Blood Services, South Africa, toward vaginal sample donation for biobanking
Source: Front Reprod Health. 2024 Nov 27;6:1446809. doi: 10.3389/frph.2024.1446809 (PMC11631888; doi:10.3389/frph.2024.1446809)
Supplement: Supplementary file 2 [file Table1.docx]

**Supplementary Table S1**: Covariates investigated as contributing factors to the willingness to donate vaginal samples.

| **Covariate** | **Description** |
| --- | --- |
| Clinic | Site at which questionnaire was collected |
| Age | Participant age at visit |
| Occupation | Categorical classification of participant occupation:   - Learner/student - Employed - Unemployed |
| Regular.blood | Do you regularly donate blood? |
| Organ.donor | Are you an organ donor / would you consider becoming an organ donor? |
| Prior.healthy | Do you have prior knowledge of the concept of a healthy vaginal microbiome? |
| Prior.transplant | Do you have prior knowledge of what a vaginal microbiota transplant is? |
| Prior.transplant.help | Do you have prior knowledge of how vaginal microbiota transplants could help patients? |
| Prior.sampled | Do you have prior knowledge of how a vaginal biome is sampled? |
| Compensation | Would you be more likely to become a vaginal sample donor if economic compensation is offered? |
| Amount | Would you consider being a vaginal sample donor if receiving the following compensation per donation:   - None - <150 ZAR (<8 USD) - >150 to <250 ZAR (>8 to <13 USD) - >250 ZAR (<13 USD) - Any amount/travel costs |
| Helping.others | Do you believe helping others is more important than any inconvenience being a vaginal donor may impose? |

**Supplementary Table S2**: Variables used to characterise the willing donors.

| **Covariate** | **Description** |
| --- | --- |
| Donor.frequency | How often would you be willing to donate vaginal samples? |
| Preferred.collection | Which of the options below would you prefer for vaginal sample collection?   - Self-collected vaginal swab - Healthcare worker-collected vaginal swab - Menstrual cup secretions (e.g., Softcup) |
| Self.collect | Where would you prefer to self-collect vaginal samples for donation? |
| Drop.off | Would you be able to commit to dropping self-collected vaginal samples off at the WCBS HQ, Pinelands, Cape Town within 24 hours of collection? |
| Amount | Would you consider being a vaginal sample donor if receiving the following compensation per donation:   - None - <150 ZAR - >150 to <250 ZAR - >250 ZAR - Any amount/travel costs |
| Affect.blood | Would donating vaginal samples affect your blood donations in any way? |
| Used.for | Would you be willing for your vaginal sample and/or the bacteria that live in it to be used for:   - Clinical purposes - Research purposes - Development of probiotic products - All of the above - None of the above |
| Reason | If you choose to become a vaginal sample donor, what would your main reason be? |
| Feedback | Would you like to know how your donations are helping patients requiring vaginal microbiota transplants? |
| Receive.transplant | If you were sick, would you be willing to receive a vaginal microbiota transplant? |

**Supplementary Table S3**: Reasons for being unwilling to donate a vaginal sample.

| **Covariate** | **Description** |
| --- | --- |
| Receive.transplant | If you were sick, would you be willing to receive a vaginal microbiota transplant? |
| N1 | Vaginal sample collection would be unpleasant |
| N2 | Vaginal sample collection would be embarrassing |
| N3 | Vaginal sample collection procedure seems too complicated |
| N4 | Vaginal sample collection would not align with cultural beliefs |
| N5 | Vaginal sample collection would be too much of a commitment to donate once a week |
| N6 | Vaginal sample collection would be too much of a commitment to donate once a month |
| N7 | Medical examinations at Western Cape Blood Service HQ, Pinelands, Cape Town during donations would be too time consuming |
| N8 | Medical examinations at Western Cape Blood Service HQ, Pinelands, Cape Town during donations would be too exhaustive |
| N9 | Do not agree with the concept of vaginal microbiota transplant procedures |
| N10 | Logistics (residing/working >2 hours from Western Cape Blood Service HQ, Pinelands, Cape Town |
